# Factors influencing the result of superior oblique weakening procedures in patients with superior oblique overaction in horizontal strabismus

**DOI:** 10.1186/s12886-020-01687-4

**Published:** 2020-10-20

**Authors:** Junwoo Chun, Seong-Joon Kim

**Affiliations:** Department of Ophthalmology, Seoul National University College of Medicine, 101 Daehak-Ro, Jongno-Gu, Seoul, 110-744 Republic of Korea

**Keywords:** Superior oblique overaction, Superior oblique weakening procedure, Dissociated vertical deviation

## Abstract

**Background:**

Few studies have evaluated the surgical outcome of superior oblique weakening procedures in patients with superior oblique overaction associated with exotropia or esotropia. This study aimed to evaluate the outcome of superior oblique muscle weakening and the influencing factors in patients with superior oblique overaction.

**Methods:**

The medical charts of 37 patients (55 eyes) with superior oblique overaction associated with esotropia or exotropia who were treated with a superior oblique weakening procedure at the Seoul National University Hospital from January 2010 to June 2017 were retrospectively reviewed. Superior oblique overaction was graded using, a 6-point scale ranging from + 0.5 to + 3, and pre- and postoperative grades were recorded for all patients.

**Results:**

The mean age of the patients was 91.81 ± 59.37 months. Superior oblique muscle suture spacer and superior oblique posterior tenectomy were performed for 17 (23 eyes) and 20 (32 eyes) patients, respectively. Surgical success was achieved in 15 (65.2%) eyes in the suture spacer group and 23 (71.9%) eyes in the posterior tenectomy group. Surgical success was achieved for 69.1% (38/55 eyes) of patients. Dissociated vertical deviation exhibited a significant negative association with the surgical success rate (*p* < 0.001).

**Conclusions:**

There was no significant difference in surgical success rate between the superior oblique posterior tenectomy and superior oblique suture spacer groups in superior oblique overaction associated with horizontal strabismus. Associated dissociated vertical deviation can affect the surgical success of the superior oblique weakening procedure.

## Background

Superior oblique weakening procedures are used to treat superior oblique overaction, A- pattern strabismus occurs due to superior oblique overaction, inferior oblique muscle palsy, and Brown syndrome. Superior oblique weakening procedures include the superior oblique muscle tenectomy, tenotomy, Z-tenotomy, silicone expander, and suture spacers.

Earlier studies have reported a 65% success rate of superior oblique lengthening using silicone expanders in Brown syndrome, 67% success of using suture spacer in Brown syndrome, and 75% success rate of superior oblique lengthening procedures with silicone expanders in A-pattern strabismus [1–3]. Other studies have reported A-pattern collapse after a superior oblique weakening procedure in 60 to 84% of patients [4–6].

However, there have been few studies on the surgical outcome of superior oblique weakening procedures for the superior oblique overaction associated with exotropia or esotropia.

Therefore, we aimed to investigate the clinical outcome and factors affecting the outcome of the superior oblique weakening procedure in patients with superior oblique overaction.

## Methods

This study was approved by the Institutional Review Board of Seoul National University Hospital in Korea. (No. 1802–082-923) This study protocol followed the tenets of Declaration of Helsinki.

The medical charts of 37 consecutive patients (55 eyes) with superior oblique overaction, who underwent a superior oblique weakening procedure (superior oblique posterior tenectomy or superior oblique suture spacer) between January 2010 and June 2017 were retrospectively reviewed. Superior oblique posterior tenectomy was performed between January 2010 and August 2013, while the superior oblique suture spacer was performed between September 2013 and June 2017. As we believed that it would be better to avoid removing part of the muscle, a suture spacer was instead considered for the surgical techniques. All surgeries were performed by the same surgeon (SJK). Patients with superior oblique overaction associated with exotropia and esotropia were included. Those with Brown syndrome and double elevator palsy were excluded. While patients with primary superior oblique overaction were included in this study, those with secondary superior oblique overaction due to inferior oblique paresis were excluded. We also excluded patients with a history of vertical rectus muscle surgery or oblique muscle surgery.

The following patient characteristics were recorded: sex, age during surgery, refractive error determined using cycloplegic refraction, angle of deviation at a distance, dissociated vertical deviation, preoperative and postoperative superior oblique overaction, and fundus torsion were assessed using fundus photography.

The prism and alternate cover test were performed at 6 m for cooperative patients, and the Krimsky test was performed for uncooperative patients.

Superior oblique overaction was graded using a 6-point scale ranging from + 0.5 to + 3. During testing for the version of both eyes, the patients were instructed to look downward and 30° laterally in both directions. When both eyes were parallel, they were considered normal and without superior oblique overaction. However, when the adducted eye was directed vertically downward, the superior oblique overaction was defined as grade 3+. The intermediate ranges were graded 1+ and + 2, respectively. A grade of + 0.5 was assigned in cases of minimal overaction.

The dissociated vertical deviation was distinguished by observation of the following: (1) characteristic elevation and excyclotorsion of the involved eye under cover and (2) absence of hypotropic movement of the other eye in any field of gaze. All measurements were made by the same observer (SJK).

Cycloplegic refraction was performed with 1% cyclopentolate hydrochloride. Refractive errors measured by cycloplegic refraction were recorded as spherical equivalents.

Ocular torsion was assessed by fundus photography, and Retcam (RetCam Clarity Medical Systems Inc., Pleasanton, CA) before the surgery. Retcam was conducted in pediatric patients who were unable to cooperate with patients laying in a supine position while under chloral hydrate-induced sedation. The amount of ocular torsion was determined by measuring the disc fovea angle (DFA), using public domain software (Image J; National Institutes of Health, Bethesda, MD). The DFA is defined as the angle between a horizontal line extending from the center of the optic disc and a line from the center of the disk to the fovea.

Stereopsis was measured using the Titmus Fly Stereo Test (Stereo Optical, Chicago, IL), in cooperative patients, before and after surgery.

All surgical procedures were conducted by the same surgeon (SJK) under general anesthesia. Surgical success was defined as the complete absence of superior oblique overaction 1 month after surgery. Failure of operation was defined as the incomplete reduction or elimination of superior oblique overaction after surgery.

We evaluated the success rate and factors influencing surgical response, by comparing the data of patients who underwent the unilateral and bilateral superior oblique muscle weakening procedures. We also compared the surgical success rate and factors, according to the procedure (posterior tenectomy, suture spacer). Finally, we compared the difference between the surgical success and failure groups. Statistical analyses were performed using SPSS Statistics 19.0 (IBM Corp., Armonk, NY). *P* < 0.05 was considered to be statistically significant. The independent t-test, chi-square test, Fisher’s exact test, and the Mann-Whitney U test were used for statistical analyses.

## Results

Among 37 consecutive patients (55 eyes) with superior oblique overaction, 19 patients (19 eyes) underwent the unilateral superior oblique weakening procedure and 18 patients (36 eyes), underwent the bilateral procedure. Seventeen patients (23 eyes) were treated with the superior oblique suture spacer approach, and 20 patients (32 eyes) were treated with superior oblique posterior tenectomy (Table 1).

**Table 1:** Surgical methods and number of patients (eyes)

	Unilateral group	Bilateral group	Total
Suture spacer group	11 (11)	6 (12)	17 (23)
Posterior tenectomy group	8 (8)	12 (24)	20 (32)
Total	19 (19)	18 (36)	37 (55)

Three patients (6 eyes) had esotropia, while 34 patients (49 eyes) had exotropia. The mean age was 91.81 ± 59.37 months. The male-female ratio was 16 (25 eyes): 21 (30 eyes).

Horizontal strabismus procedure was successful in 31 patients (83.8%) while failure was reported in 6 patients (surgical success was defined when values ≤5 and ≤ 10 prism were observed esotropia and exotropia, respectively).

There was no significant correlation with between successful horizontal strabismus procedure and superior oblique weakening procedure (*p* = 0.162, Fisher’s exact test).

Similary, no significant differences in patient characteristics and surgical success rate were found between the superior oblique posterior tenectomy and superior oblique suture spacer groups (Table 2).

**Table 2:** Comparison according to the method of superior oblique weakening

	Total	Suture spacer	Posterior tenectomy	*P*-value
Sex (M: F) (*N* = 37)	16: 21	7: 10	9: 11	0.815
Age at operation (months) (*N* = 37)	91.81 ± 59.37	84.06 ± 41.24	98.40 ± 71.74	0.472
Associated horizontal strabismus (PD) (*N* = 37)	20.19 ± 18.98	18.35 ± 18.07	21.75 ± 20.04	0.595
Surgical success (success: fail)	38: 17	15: 8	23: 9	0.598
Refractive error (spherical equivalent)	0.36 ± 2.34	0.20 ± 2.13	0.48 ± 2.50	0.656
DVD (positive: negative)	23: 32	9: 14	14: 18	0.732
Preoperative SO overaction (+ 0.5 to + 3)	1.59 ± 0.47	1.83 ± 0.32	1.42 ± 0.49	0.002^a^
Torsion (−: intorsion, +: extorsion) degree (*N* = 43)	−1.23 ± 6.38	−0.32 ± 6.69	− 1.50 ± 7.64	0.597

*PD* prism diopters, *SO* superior oblique, *DVD* dissociated vertical deviation

^a^*p* < 0.05 Mann-Whitney test

In contrast, the occurrence of ocular incyclotorsion significantly differed between the unilateral and bilateral groups (*p* = 0.013). However, the groups did not differ significantly in any other factors, such as type of surgical procedure, age during surgery, refractive error, dissociated vertical deviation, preoperative superior oblique overaction, and associated horizontal strabismus (either exotropia or esotropia). Moreover, no difference was identified in the surgical success rate between the two groups (Table 3).

**Table 3:** Comparison of the unilateral group and bilateral group

	Total	Unilateral group	Bilateral group	*P*-value
Sex (M: F) (N = 37)	16: 21	7: 12	9: 9	0.419
Age at operation (months) (*N* = 37)	91.81 ± 59.37	79.63 ± 46.26	104.67 ± 68.70	0.204
Associated horizontal strabismus (PD) (*N* = 37)	20.19 ± 18.98	20.89 ± 17.85	19.44 ± 20.60	0.820
SO weakening procedure (suture spacer: posterior tenectomy)	23: 32	11: 8	12: 24	0.079
Surgical success (success: fail)	38: 17	13: 6	25: 11	0.938
Refractive error (spherical equivalent)	0.36 ± 2.34	0.05 ± 1.46	0.53 ± 2.70	0.400
DVD (positive: negative)	23: 32	7: 12	16: 20	0.587
Preoperative SO overaction (+ 0.5 to + 3)	1.59 ± 0.47	1.60 ± 0.43	1.58 ± 0.50	0.866
Torsion (−:intorsion, +:extorsion) degree (*N* = 43)	−1.23 ± 6.38	3.08 ± 7.00	−2.74 ± 6.60	0.013^a^

*PD* prism diopters, *SO* superior oblique, *DVD* dissociated vertical deviation

^a^*p* < 0.05 independent t-test

There were no significant differences in any variable except dissociated vertical deviation between the surgical success and failure groups. There was a statistically significant difference in the dissociated vertical deviation (*p* < 0.001) between the two groups, and the rate of surgical success decreased with an increase in dissociated vertical deviation (Table 4).

**Table 4:** Comparison of the success group and failure group

	Total	Success group	Failure group	*P*-value
Sex (M: F) (N = 37)	16: 21	11: 16	5: 5	0.716
Age at operation (months) (*N* = 37)	91.81 ± 59.37	96.10 ± 38.95	90.22 ± 65.93	0.793
Associated horizontal strabismus (PD) (N = 37)	20.19 ± 18.98	24.51 ± 7.75	16.58 ± 3.19	0.291
SO weakening procedure (suture spacer: posterior tenectomy)	23: 32	15: 23	8: 9	0.598
Refractive error (spherical equivalent)	0.36 ± 2.34	0.51 ± 3.23	0.30 ± 1.86	0.797
DVD (positive: negative)	23: 32	10: 28	13: 4	< 0.001^a^
Preoperative SO overaction (+ 0.5 to + 3)	1.59 ± 0.47	1.51 ± 0.47	1.76 ± 0.44	0.670
Torsion (−: intorsion, +: extorsion) degree (*N* = 43)	−1.23 ± 6.38	0.12 ± 7.29	−3.26 ± 6.59	0.150

*PD* prism diopters, *SO* superior oblique, *DVD* dissociated vertical deviation

^a^*p* < 0.05 chi-square test

Stereopsis was measured for 16 of 37 patients at 1 month before and 1 month after the surgery. Ten patients (62.5%) showed an increase of more than two octaves, while the remaining six (37.5%) showed a change lower than two octaves.

## Discussion

Superior oblique posterior tenectomy and the superior oblique muscle suture spacer approach are widely used procedures for superior oblique weakening [7]. Earlier studies have suggested that they are effective in weakening superior oblique overaction and also for correcting the A-pattern [4, 8].

The surgical success rate of the superior oblique weakening procedure was 69.1% (38/55 eyes) in this study. Although the improvements in the A-pattern after surgery were not investigated, we evaluated the superior oblique overaction with detailed grading at duction and version.

Superior oblique weakening procedures cannot guarantee binocular vision due to the ocular torsion after surgery for the superior oblique overaction associated with exotropia or esotropia. However, our study showed that 62.5% (10/16) of patients experienced an increase in stereopsis and no patient showed a decrease in stereopsis.

The normal range of DFA is 5.6° - 8° [9–13]. DFA was 3.08° ± 7.00° in the unilateral group, indicative of mild incyclotorsion. However, it was − 2.74° ± 6.60° in the bilateral group, indicative of greater incyclotorsion, which is evident of superior oblique overaction. Incyclotorsion was naturally greater in the bilateral group.

There were no significant differences in the patient characteristics and surgical success rates between the superior oblique suture spacer and posterior tenectomy groups. Suture spacers are generally thought to have a greater surgical effect, but there were no difference. There were no significant differences in any variable except the dissociated vertical deviation between the surgical success and failure groups.

The cause of dissociated vertical deviation is unknown. Several etiological theories have been proposed, including superior rectus hypofunction, anomalous impulses from an involuntary divergence center, aberrations of postural tonus mechanisms of the extraocular musculature, bilateral paresis of the depressor muscles, and deficiency in the optomotor impulse from the inferonasal retinal quadrants. Dissociated vertical deviation may also result from superior oblique hypofunction or an alternating innervational insufficiency of the superior oblique muscles [14–16]. According to Wright et al. [17], superior oblique weakening procedures can cause complications in patients with preexisting dissociated vertical deviation, as the weakening of the superior oblique can exacerbate dissociated vertical deviation.

In our study, the success rate of the superior oblique weakening procedures significantly decreased with the presence of dissociated vertical deviation. Superior oblique overaction accompanied with dissociated vertical deviation seems to have a different etiology than superior oblique overaction alone. Although the cause of superior oblique overaction in patients with horizontal strabismus and the cause of accompanying dissociated vertical deviation are unknown. It can be assumed that surgical outcomes could worsen when superior oblique overaction and dissociated vertical deviation coexist.

Our study has a few limitations. First, this was a retrospective study. Second, the A-pattern was not evaluated. Third, Retcam, rather than fundus photography, was used for noncompliant pediatric patients. To the best of our knowledge, the cyclotorsion induced by choral hydrate has not been reported ever. However, given that cyclotorsion was possible in the supine position [18], the failure to control for this variable was included as a limitation of this study. An internal fixator was not used for accurate measurement of ocular torsion and postoperative torsional change was not evaluated. Finally, the effect of horizontal strabismus surgery on the surgical result of superior oblique weakening procedure was not evaluated.

## Conclusions

To the best of our knowledge, this is the first study, in which the superior oblique weakening procedure was evaluated in terms of duction and version. Associated dissociated vertical deviation can affect the surgical success of the superior oblique weakening procedure.

## Data Availability

The datasets used and/or analysed during the current study available from the corresponding author on reasonable request.

## Abbreviation

DFA: Disc fovea angle
